# Spatial Patterns in Biofilm Diversity across Hierarchical Levels of River-Floodplain Landscapes

**DOI:** 10.1371/journal.pone.0144303

**Published:** 2015-12-02

**Authors:** Marc Peipoch, Ryan Jones, H. Maurice Valett

**Affiliations:** 1 Division of Biological Sciences, University of Montana, Missoula, Montana, United States of America; 2 Department of Microbiology and Immunology, Montana State University, Bozeman, Montana, United States of America; INRA, FRANCE

## Abstract

River-floodplain systems are among the most diverse and productive ecosystems, but the effects of biophysical complexity at multiple scales on microbial biodiversity have not been studied. Here, we investigated how the hierarchical organization of river systems (i.e., region, floodplain, zone, habitats, and microhabitats) influences epilithic biofilm community assemblage patterns by characterizing microbial communities using 16S rRNA gene sequence data and analyzing bacterial species distribution across local and regional scales. Results indicate that regional and local environmental filters concurrently sort bacterial species, suggesting that spatial configuration of epilithic biofilms resembles patterns of larger organisms in floodplain ecosystems. Along the hierarchical organization of fluvial systems, floodplains constitute a vector of maximum environmental heterogeneity and consequently act as a major landscape filter for biofilm species. Thus, river basins and associated floodplains may simply reflect very large scale ‘patches’ within which environmental conditions select for community composition of epilithic biofilms.

## Introduction

River-floodplain landscapes are characterized by shifting habitat mosaics [1] and are governed by flood pulses that link terrestrial and aquatic reservoirs [2, 3]. This unique combination of habitat heterogeneity and variable hydrologic connectivity enhances variation in factors controlling species diversity such as disturbance [4], resource availability [5], and edge effects associated with emergent ecotones [6, 7]. The fundamental argument for floodplains recognition as among the most diverse and productive ecosystems world-wide [8] is that their exceptional biophysical complexity at the landscape scale provides a plethora of niches for both aquatic and terrestrial species [1]. Niche-based assessments of floodplain diversity, however, have almost exclusively focused on “large” organisms like invertebrates, fish, or vascular plants [9–11].

Based heavily on the historical premise that ‘everything is everywhere, but the environment selects’ [12], microbial ecologists have implicitly relied on niche-based arguments to explain patterns in microbial species distribution [13, 14]. Accordingly, microbial community assembly has generally been considered to be organized by local environmental controls (i.e., species sorting at the habitat scale), or at least local influences are more easily manifested than those associated with larger-scale factors like dispersal [15, 16]. With this perspective, biogeographic patterns of microbial species are not particularly affected by geographic distances or historic events and instead exhibit greater local:global richness ratios than those of larger organisms [17, 18]. Some recent studies, however, argue that patterns in microbial biogeography can be similar to those of larger organisms. Hillebrand and Blenckner [19] and Green and Bohannan [20], assert that both local (i.e., environmental) and regional (i.e. dispersal, legacies) factors influence microbial composition, and predict more gradual decreases in microbial diversity from larger to more local scales. From this perspective, species from a regional pool must first “pass” through a series of nested filters (i.e., scaled habitat features that influence the probability that the taxon with its specified traits is able to join as members of a local community, [18]) before ultimately contributing to microbial assembly at the local scale [21].

**Fig 1:**
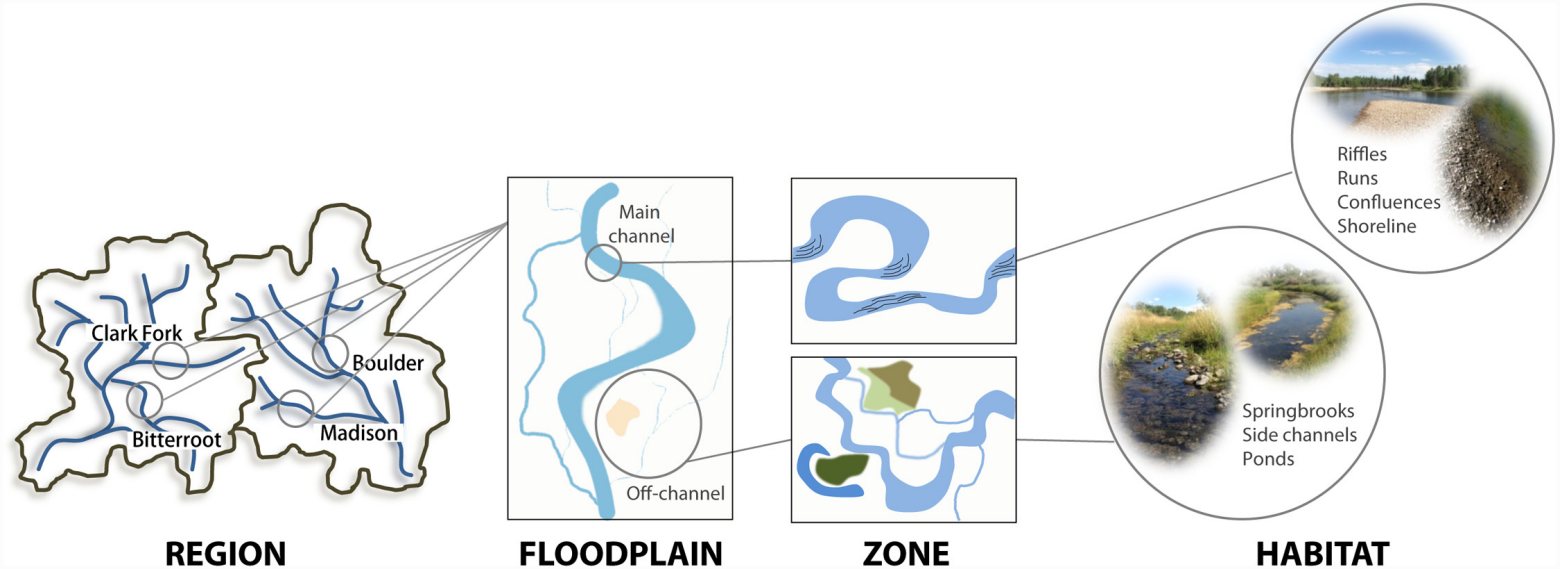
Hierarchical organization of river-floodplain systems. Proposed structural units along the hierarchical organization of river systems.

Fluvial systems embody an inherent hierarchical organization from catchment to habitat scales [22], which provides an ideal perspective to examine both environmental and spatial controls on microbial communities across multiple scales. With focus on floodplain ecosystems, the framework we employ herein recognizes five different structural units, or holons [23], that can be broadly distinguished along the hierarchical organization of river-floodplain systems (Fig 1 & S1 Table) including: 1) regions, physiographic entities that define geographic areas of generally similar climatic and hydrologic conditions, 2) floodplains, geomorphic surfaces intimately associated with rivers and the largest spatial entity at which discernable environmental features are identified, 3) zones, sub-systems reflecting spatial variation in aquatic-terrestrial interaction, 4) habitats, specific small-scale channel units (e.g., riffles, runs, shoreline, ponds, etc.) that are the smallest scale at which environmental character is discerned, and 5) microhabitats, sub-habitat niche space that causes variation within a given habitat type.

Here, we employ this hierarchical organization of river-floodplain systems with the recognition that patterns at one level reflect mechanisms characteristic of the level below in order to assess how regional and local features may interact to dictate bacterial species abundance and distribution among epilithic biofilms, the most ubiquitous microbial biotope in the benthos of gravel-bed floodplains. To do this, we combine large-scale biophysical surveys of four river systems in western Montana, USA, with 56 measures of microbial community structure derived from 16S rRNA gene composition.

## Materials and Methods

### Field sampling

The study was conducted during late July 2013 in the Bitterroot (46.6920, -1140445), Clark Fork (46.6224, -113.0781), Boulder (46.1460, -111.9836), and Madison Rivers (45.7772, -111.5122) of western Montana, USA. Most of the study sites were located in public lands and did not required specific permissions to access and sample the river and floodplain ecosystems. For those sites located in private lands, owners were contacted and they gave permission to access and sample both river and floodplain environments. This study did not involve endangered or protected species. These four rivers have snowmelt-driven with spring flooding that creates and sustains relatively well-preserved, gravel-bed floodplains. The Bitterroot River drains a catchment of 6066 km^2^ with a historical mean annual discharge of 60.3 ± 3.2 m^3^ s^-1^; at the time of this study, mean water depth and velocity in its main channel were 0.8 m and 0.3 m s^-1^, respectively. Despite the relatively similar catchment area at our sampling locations, the Clark Fork (4595 km^2^) and Madison (6474 km^2^) Rivers show a very different annual discharge of 14.7 ± 0.8 m^3^ s^-1^ and 49.5 ± 1.1 m^3^ s^-1^, respectively. The Clark Fork River was mainly characterized by shallow, slow-moving water, 0.1 m and 0.01 m s^-1^; while the Madison River showed consistently multiple channels across the floodplain but also a deep, fast main channel (2.4 m and 0.4 m s^-1^). The Boulder River was by far the smallest river-floodplain system included in this study, with a catchment area of 987 km^2^ and an annual discharge of 3.4 ± 0.3 m^3^ s^-1^. At the time of the study, water depth and velocity in the Boulder main channel were 0.3 m and 0.6 m s^-1^, respectively. For each river, a ca. 9-km reach was selected and equally divided among upper, middle, and lower sections. For each section, habitats within main channel (riffles, runs, channel confluences, shorelines) and off-channel zones (side channels, ponds, parafluvial springbrooks, orthofluvial springbrooks) were sampled for a total of 56 samples from eight habitats (S1 Table). Geographic locations were determined by GPS (eTrex 20 [Garmin, Salem, Oregon, USA]), environmental conditions measured (16 biophysical variables, S2 Table), and biofilm community structure characterized as below.

Surface water samples were collected in triplicate at each habitat, filtered in the field (Whatman GFF with 0.7 μm average particle size retained [Whatman International, Kent, UK]), and frozen until analyzed for nutrients (e.g., inorganic nitrogen (N) and phosphorus (P)) and other water quality parameters, S2 Table). Epilithic samples for chlorophyll and organic matter (ash-free dry mass, AFDM) were collected as composites scrapes from three cobbles of known area within each habitat and stored on ice until processed in the laboratory. We also collected composite samples into 2-mL Safe-Lock microcentrifuge tubes (Eppendorf AG, Germany) from 15 to 20 different cobbles by scraping epilithic biofilms using sterile disposable spatulas. Samples were centrifuged (12500 rpm, 10 min), water excess removed, and biofilm samples stored at -20°C until processed for microbial community composition.

### DNA extraction and phylogenetic analysis

Deoxyribonucleic acid (DNA) was extracted from epilithic biofilms using the PowerBiofilm^®^ DNA Isolation Kit (MO BIO Lab. Inc., Carlsbad, California, USA) following the manufacturer’s instructions. The V4 region of the16S rRNA gene was amplified in triplicate using primers F515/R806 tagged with 12-base Golay codes [24]. Amplicons were sequenced using the Illumina MiSeq platform at Argonne National Laboratory. Approximately 250 base-pair reads were generated from each direction, resulting in nearly the entire targeted region having double coverage. Reads were assembled, Organizational Taxonomic Units (OTUs) were generated based on 97% sequence similarity, and chimeras were removed using USEARCH v7.0 [25, 26]. To optimize diversity characterizations among samples, the entire dataset was rarefied to 17,000 DNA sequences per sample using QIIME v1.8 [27]. Similarly, bacteria and cyanobacteria datasets were rarefied to 9,000 and 100 DNA sequences per sample, respectively. The final dataset included 8,826 OTUs from 56 individual biofilm samples. Representative DNA sequences for each OTU were aligned using PyNAST as implemented in QIIME [28] and filtered using the 16S Lane mask (see mapping file used for QIIME analysis in S3 Table). Taxonomy of each representative sequence was assigned based on Ribosomal Database Project taxonomy scheme (http://rdp.cme.msu.edu).

### Statistical analysis

Variation in biofilm, bacteria, and cyanobacteria community composition at each hierarchical level (i.e., region, floodplain, zone, and habitat) was examined using principal coordinate analysis (PCoA) conducted with both Bray-Curtis and weighted UniFrac distance matrices using *cmdscale* function in R’s “stats” package. Variation in the relative abundance of different bacteria phyla associated with river, zone, and habitat levels was calculated using a variance component model with river, zone (nested within river), and habitat (nested within zone) as random factors. Analysis of similarity (ANOSIM, R’s *anosim* function) based on both Bray-Curtis and UniFrac distances was then performed to assess differences among groups within each hierarchical level. Measures of biodiversity included Shannon-Wiener index and beta diversity (Sorensen’s index of dissimilarity) using *betadiver* function in R’s “betadiver” package. Diversity metrics for each level of the hierarchy were derived only from replicate entities extant at the next lower level (e.g., regional patterns were derived from comparisons among floodplains, n = 4). Comparisons at the habitat scale reflect within habitat variation as provided by replicate measures within each floodplain and zone. Redundancy analysis was employed to explore multivariate relationships between environmental parameters and biofilm composition across different river-floodplain systems using *cca* function in R’s “vegan” package. Partial Mantel tests were used to compare OTUs similarity to environmental distances while controlling for geographic distances, and vice versa (R *mantel* function). When environmental control was significant, we performed single Mantel correlations between community structure and Euclidean distances of individual environmental variables to evaluate environmental influences on bacteria and cyanobacteria assemblies. All statistical tests were done using R 3.1.3 (R Foundation for Statistical Computing, Vienna, Austria. http://www.R-project.org/).

## Results and Discussion

### Community composition: hierarchical comparisons

**Fig 2:**
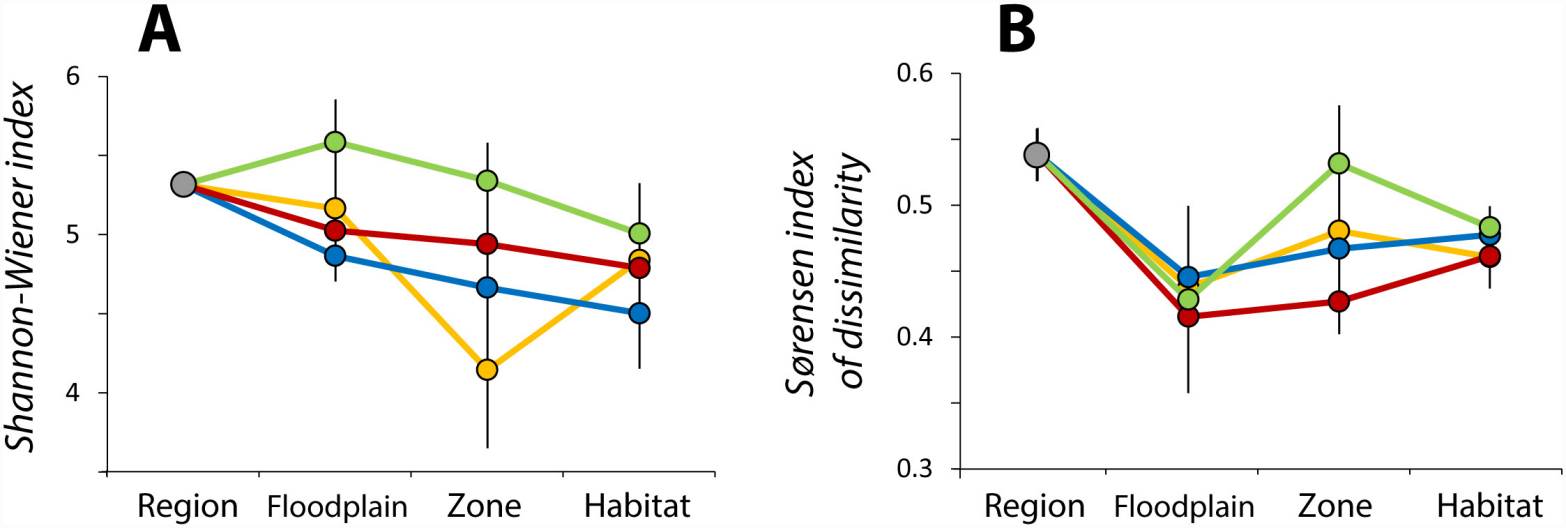
Biofilm diversity patterns across the hierarchical organization of river-floodplain systems. Data points for each river-floodplain system represent mean values ± SEM of Shannon-Wiener (A) and beta-diversity (B) indices of biofilm communities at each hierarchical level. Diversity indices for each specific identity at each hierarchical level were calculated from group-averaged OTUs abundances within the next lower level (e.g., diversity indices at the floodplain level are calculated using zone-specific OTUs abundances). Prior to diversity indices calculations, 1000 bootstrap iterations were applied to normalize the number of sequenced samples per river at 10. Results associated with the Clark Fork River (orange), Boulder River (blue), Bitterroot River (red), and Madison River (green) are shown separately.

Bacterial diversity in epilithic biofilms generally decreased from upper to lower hierarchical levels of river-floodplain landscapes, with the exception of the Madison River in which diversity index was highest at the floodplain scale due to the extraordinary species diversity found in its off-channel zone (Fig 2). Biofilm β-diversity declined substantially between regional and floodplain levels, but then increased for the two remaining hierarchical levels (Fig 2). These results indicate highest variation in biofilm diversity among the four different floodplains (i.e., regional level), intermediate at the habitat and microhabitat scales, and lowest variation in biofilm diversity at the zone level.

**Fig 3:**
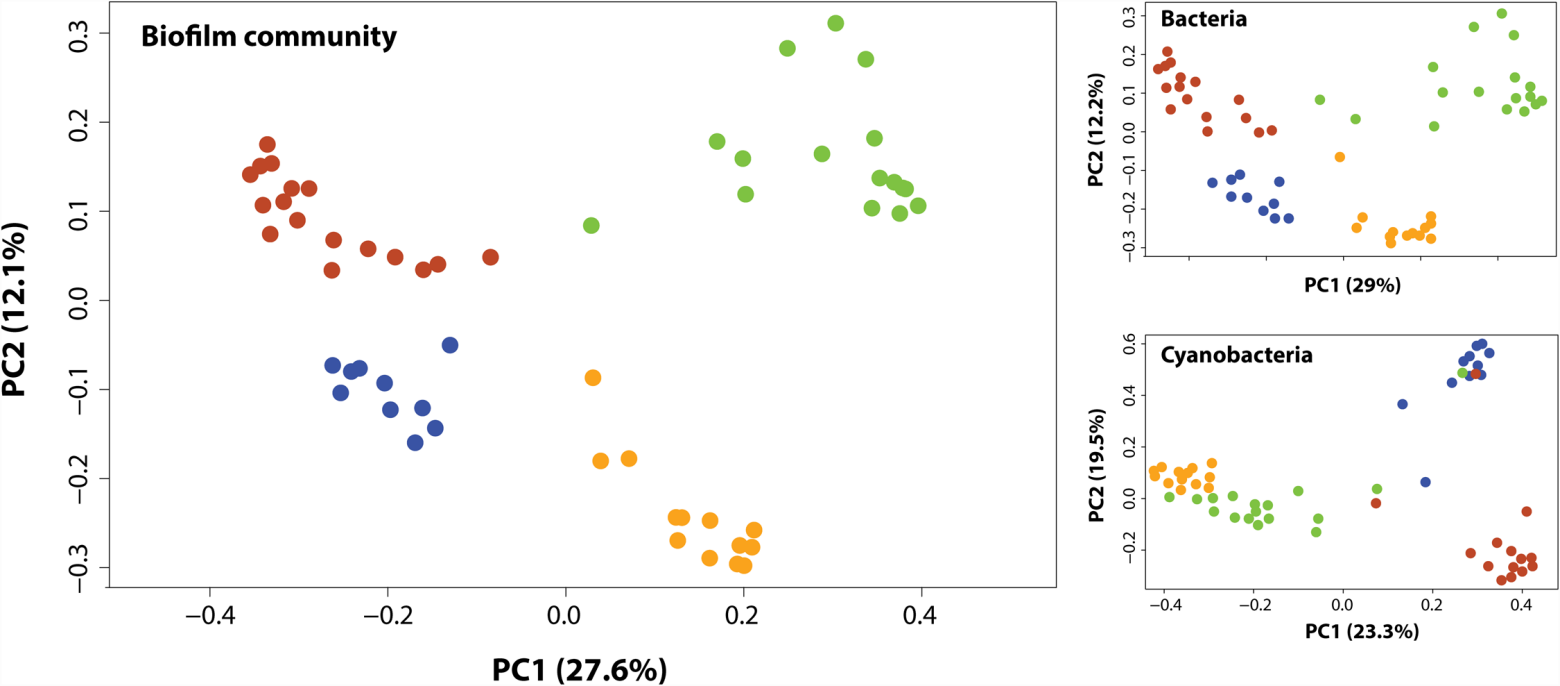
Variation in biofilm community composition at the floodplain level. Principal Coordinates Analysis (PCoA) of biofilm (i.e., bacteria and cyanobacteria), bacteria, and cyanobacteria community assemblies (based on Bray–Curtis distances among OTUs abundances) as related to the four different riverine floodplains (Color coding as in Fig 2). Percentage of the diversity distribution explained by each axes is indicated on the figure.

Floodplain-level organization of biofilm assemblies was further illustrated by a strong separation of samples based on the different river-floodplain systems (PCoA, Fig 3); no clustering occurred at any other hierarchical level. Greater structural and phylogenetic dissimilarity of biofilm communities occurred among floodplains compared to within floodplain systems based on Bray-Curtis (ANOSIM’s R: 0.76; *P* = 0.001) and weighted UniFrac (ANOSIM’s R: 0.63; *P* = 0.001) dissimilarities, respectively. Distinction among floodplains was equally evident (Fig 3) when bacterial (ANOSIM’s R: 0.75; *P* = 0.001) and cyanobacterial (ANOSIM’s R: 0.82; *P* = 0.001) constituents were analyzed separately. All pairwise comparisons of biofilm, bacteria, and cyanobacteria communities among floodplains revealed significantly different compositions (*P* < 0.001). These results suggest strong filtering of biofilm species occurring at the floodplain level. Greater environmental variation across space drives stronger species sorting via spatially-explicit processes [29]. Here, 13 of 16 environmental variables differed significantly among the four systems (S2 Table) and while environmental differences between zones and among habitats occurred (data not shown), they were of far less influence (i.e., ANOSIM analysis showed no clustering at these hierarchical levels) than the greater variation observed at the floodplain scale. The significant link between environmental variation and biofilm composition at the regional scale was further evidenced by floodplain clustering in a redundancy analysis (S1 Fig). Biofilm communities from river-floodplain landscapes with the most similar environmental conditions (i.e., Boulder and Bitterroot Rivers; S2 Table), were mixed and separated from the other two floodplain systems (S1 Fig). This pattern was also observed in the results of PCoA, in which Bitterroot and Boulder Rivers were in opposition on the first axis with Clark Fork and Madison Rivers (Fig 3).

Together, these cross-scale patterns highlight floodplains as the structural unit at which sorting and turnover of bacterial species in epilithic biofilm is principally occurring. Only 14% of total OTUs identified were present in all four floodplains, while ~25% of identified OTUs in each floodplain were unique of that particular river system (S2 Fig). Floodplains within a region, therefore, seem to constitute a key holon for the assembly of biofilm communities along the hierarchical organization of river-floodplain landscapes employed herein.

Local:global species richness ratios for biofilm communities are expected to be greater than those for large organisms according to the more classical view that these assemblages are not dispersal limited [18]. Our habitat:regional ratios averaged 0.2 ± 0.01 among the different river systems and were not only not greater, but slightly lower than those of gastropods (0.24), amphibians (0.35), and fish (0.25) observed by Ward et al. [10] in river-floodplain landscapes from the Alps region. Lower local:global species ratios, combined with a relatively gradual decrease in biofilm diversity from regional to habitat scales (Fig 2) fail to support the historical premise of microbial ubiquity and local sorting. Instead, they agree with those studies suggesting that both regional (e.g., dispersion, environmental legacies) and local (i.e., environmental) factors influence the assembly of microbial communities [20, 30]. This contention is further supported by a significant correlation between average habitat richness and floodplain richness observed in our study (r^2^ = 0.99, *p*-value<0.01, n = 4) suggesting dependence of local diversity on regional and historical processes [31].

**Fig 4:**
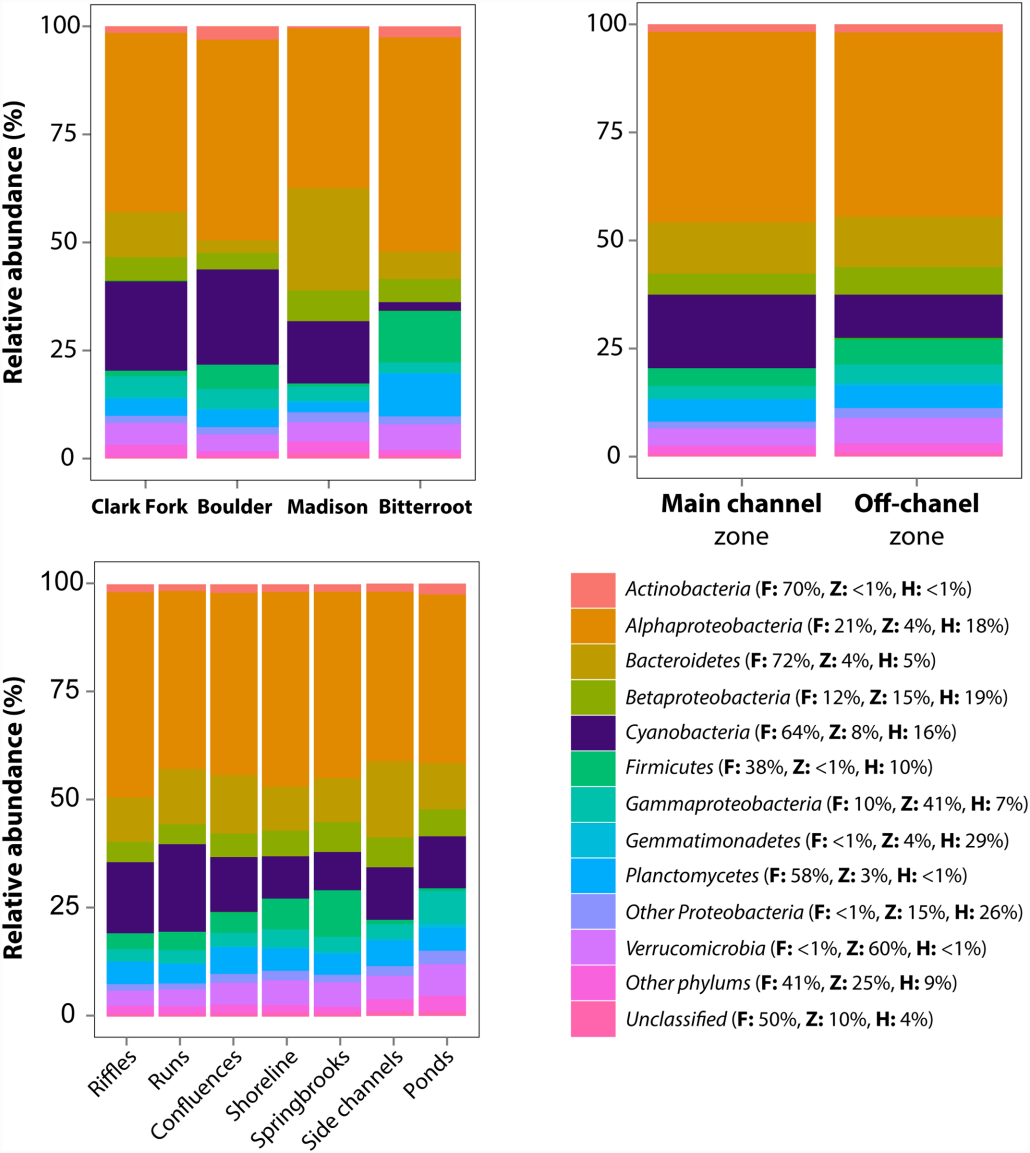
Bacterial phyla composition of biofilm communities along the hierarchical organization of river-floodplain systems. Relative abundance of bacteria phyla (class for Proteobacteria) of biofilm communities at each river system (upper-left panel), zone (upper-right panel), and habitat (lower-left panel). Variation in the relative abundance of bacteria phyla/class associated with each spatial scale: floodplain (F), zone (Z), and habitat (H) is indicated within parenthesis for each phyla/class (residual variance of the models is not indicated).

**Fig 5:**
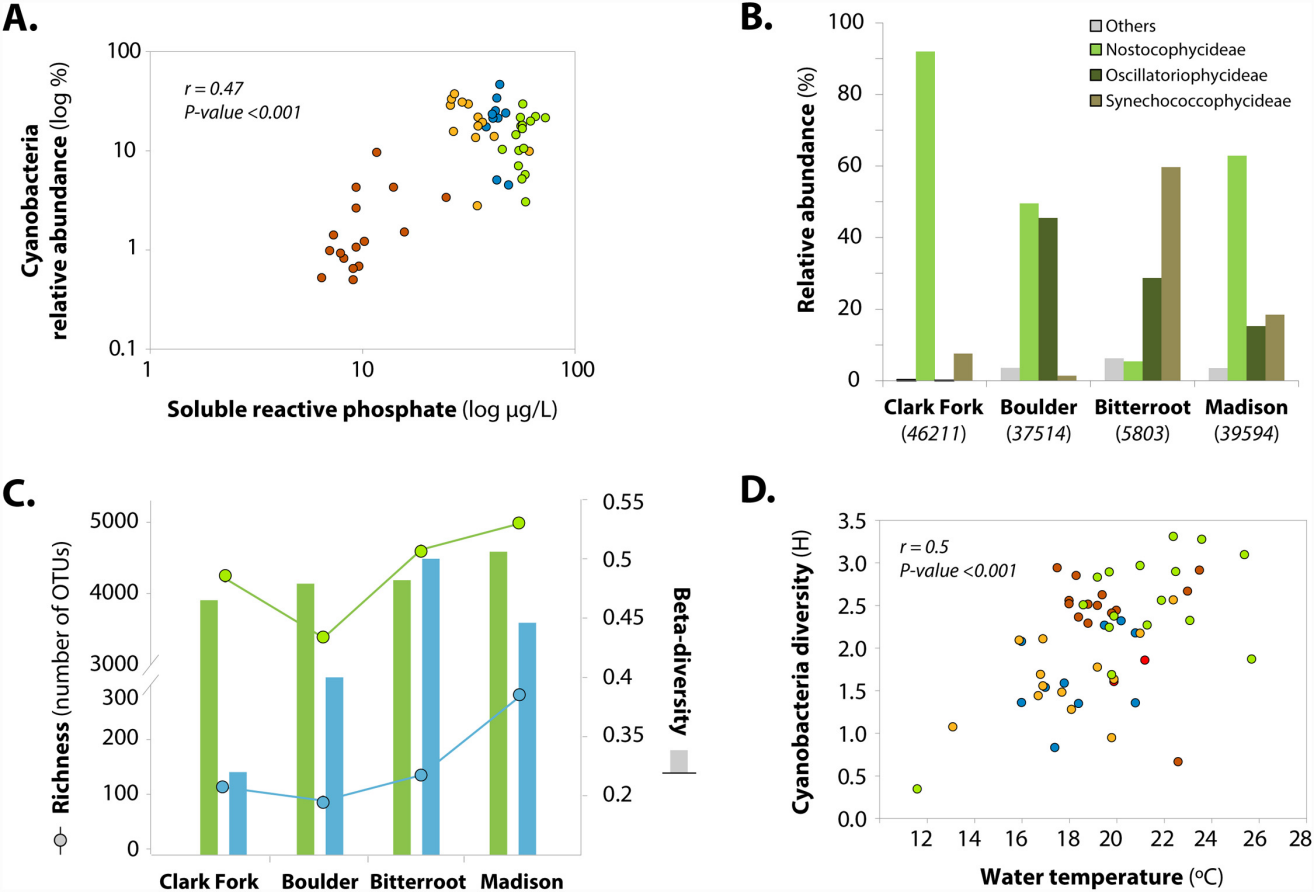
Controls on cyanobacteria communities within and between floodplains. A) Linear correlation between SRP concentrations and relative abundance of cyanobacteria (note log axis), color coding as in Fig 2. B) Relative abundance of cyanobacteria classes identified within each river system; total amount of cyanobacteria sequences per river system is shown below each floodplain’s label. C) Bars and lines represent beta-diversity values and richness, respectively, for biofilm (green) and cyanobacteria (blue) at each river system. D) Linear correlation between water temperature and species diversity of cyanobacteria, color coding as in Fig 2.

Overall, 8263 OTUs (94% of the total identified OTUs) could be assigned to 41 different bacterial phyla at a 90% confidence threshold. Most abundant OTUs were allocated to 9 phyla (S4 Table), whose relative abundance showed generally greater variation among floodplains and habitats than at the zone level (Fig 4). *Alphaproteobacteria* accounted for almost half of total abundance in biofilms from each particular floodplain, zone, and habitat; while showing similar spatial variation at floodplain and habitat scales (Fig 4). *Gemmatimonadetes*, *Betaproteobacteria*, and other *Proteobacteria* classes were the three groups with most of the variation in their relative abundance associated with the habitat scale, although they did not represent a substantial contribution to biofilm communities (Fig 4). Similarly, relative abundances of *Cyanobacteria* and *Firmicutes* did also show significant variation among habitats, but following opposite patterns (Fig 4). A number of chloroplasts of eukaryotic algae (*Stramenopiles*, *Chlorophyta*, and *Rhodophyta* orders) were identified in biofilm communities showing significant differences among floodplains (ANOSIM’s R: 0.395; P = 0.001) and zones (ANOSIM’s R: 0.07; P = 0.004). Among-floodplain variation in composition was directly reflected in differences in the relative abundance of cyanobacteria (Fig 4). Very low N concentrations during summer and corresponding low atomic N:P (0.5–4.4, S2 Table) suggest potential N-limitation of primary production across all four systems [32,33], but the relative abundance of cyanobacteria varied from less than 1% (Bitterroot) to over 20% (Clark Fork) despite low N availability (Fig 4). Observed differences in relative abundance of cyanobacteria corresponded to P availability, which varied by over an order of magnitude among floodplains (Fig 5A). Beyond their relative abundance, dominant cyanobacteria groups varied strikingly among the four river-floodplain systems (Fig 5B). In more P-rich systems, cyanobacteria were dominated by class *Nostocophycidae*, which includes heterocystous cyanobacteria with the capacity to fix atmospheric N_2_, observed as dominant in other high P-low N running waters [34, 35]. In general, microbial diversity of biofilms as a whole mirrored patterns of cyanobacteria diversity measures (Fig 5C), but were not correlated to bacterial richness. In turn, cyanobacterial diversity (as Shannon-Weiner index) was higher in habitats showing higher water temperature (Fig 5D). Temperature dependence of cyanobacteria diversity exemplifies local environmental control on biofilm communities within each floodplain since water temperature varied principally among habitats but not between floodplains (S2 Table). Conversely, P concentrations varied among floodplains but not habitats, highlighting joint influences of local and regional controls over cyanobacteria communities.

### Distinguishing global and local control: interpretations and implications

Unequivocally identifying the character of regional effects is problematic. Lindstrom and Langenheder [30] noted that although regional factors are mostly estimated using spatial distances between local sites, it is unclear whether they represent purely spatial effects (e.g., dispersal limitation) or legacies of past environmental conditions. Partial Mantel analysis (i.e., variation partitioning on distance matrices), however, is considered a robust test of the relative influence of geographic distances and environmental variables [36–38].

Within each river-floodplain system, biofilm, bacteria, and cyanobacteria composition appeared tied to environmental conditions (i.e., distances) when controlling for geographic effects (partial Mantel correlations, r(SE,G), Table 1) while no significant correlations were observed between geographic distances and OTU similarity after ruling out environmental effects (r(G,SE), Table 1). Local sorting reflects variation in physicochemical conditions such as dissolved oxygen, specific conductivity, water temperature, dissolved organic carbon, and epilithic organic matter (S5 Table); which are directly or indirectly governed by the flood pulse, a hydro-ecological phenomenon that leads to environmental and biotic heterogeneity across river-floodplain systems [1, 2]. Thus, at the habitat scale, responses to environmental variation generate unique biofilm communities within each floodplain system regardless of habitat proximity. It is worth pointing out, however, that local environmental sorting of biofilm communities observed in this study correspond to a specific time over biofilm community succession, in which environmental and biological controls on biofilm structure and composition can vary as a function of biofilm development [39]. Future research should address the role of temporal variation in local sorting of biofilm communities to further understand how environmental filtering of biofilm species at the local scale may change as the biofilm matures.

**Table 1:** Partial Mantel correlations between community structure, geographical distances, and environmental variables.

		Biofilm		Bacteria		Cyanobacteria	
	*N*	r(SG.E)	r(SE.G)	r(SG.E)	r(SE.G)	r(SG.E)	r(SE.G)
Within floodplains							
Clark Fork	*13*	0.059	-0.603**	-0.093	-0.701***	-0.068	-0.293*
Boulder	*10*	0.075	-0.454**	0.026	-0.399**	0.073	-0.801**
Bitterroot	*16*	-0.029	-0.610***	-0.014	-0.605***	-0.141	-0.433*
Madison	*17*	0.072	-0.681***	0.056	-0.555**	0.032	-0.614***
Among all floodplains	*56*	-0.013	-0.655***	-0.028	-0.671***	-0.101**	-0.435***

The partial Mantel statistic r(SG.E) estimates the Spearman rank correlation between S (OTU similarity) and G matrices (geographical distance) while controlling for the effect of environmental distances (E). Likewise, r(SE.G) calculates the Spearman rank correlation between matrices S (OTU similarity) and E (environmental distance) while controlling for the effect of geographical distance (G). Geographical matrix (G) contained spatial distances among floodplain samples. Significance was calculated from 99999 randomized permutations of one of the dissimilarity matrices (*P-value<0.05, **P-value<0.01, ***P-value<0.001).

Despite evident local environmental control on biofilm community assembly within each floodplain, the largest differences in both environmental conditions (S2 Table) and community diversity (Figs 3 and 4) were found among floodplain systems (i.e., regional effects), and patterns in biofilm diversity across hierarchical levels (Fig 2) suggest concomitant local and regional influences on the assembly of biofilm communities. Local sorting seems clearly tied to environmental variation (Table 1); the query is to discern whether co-occurring regional effects are spatially or environmentally driven [30].

**Fig 6:**
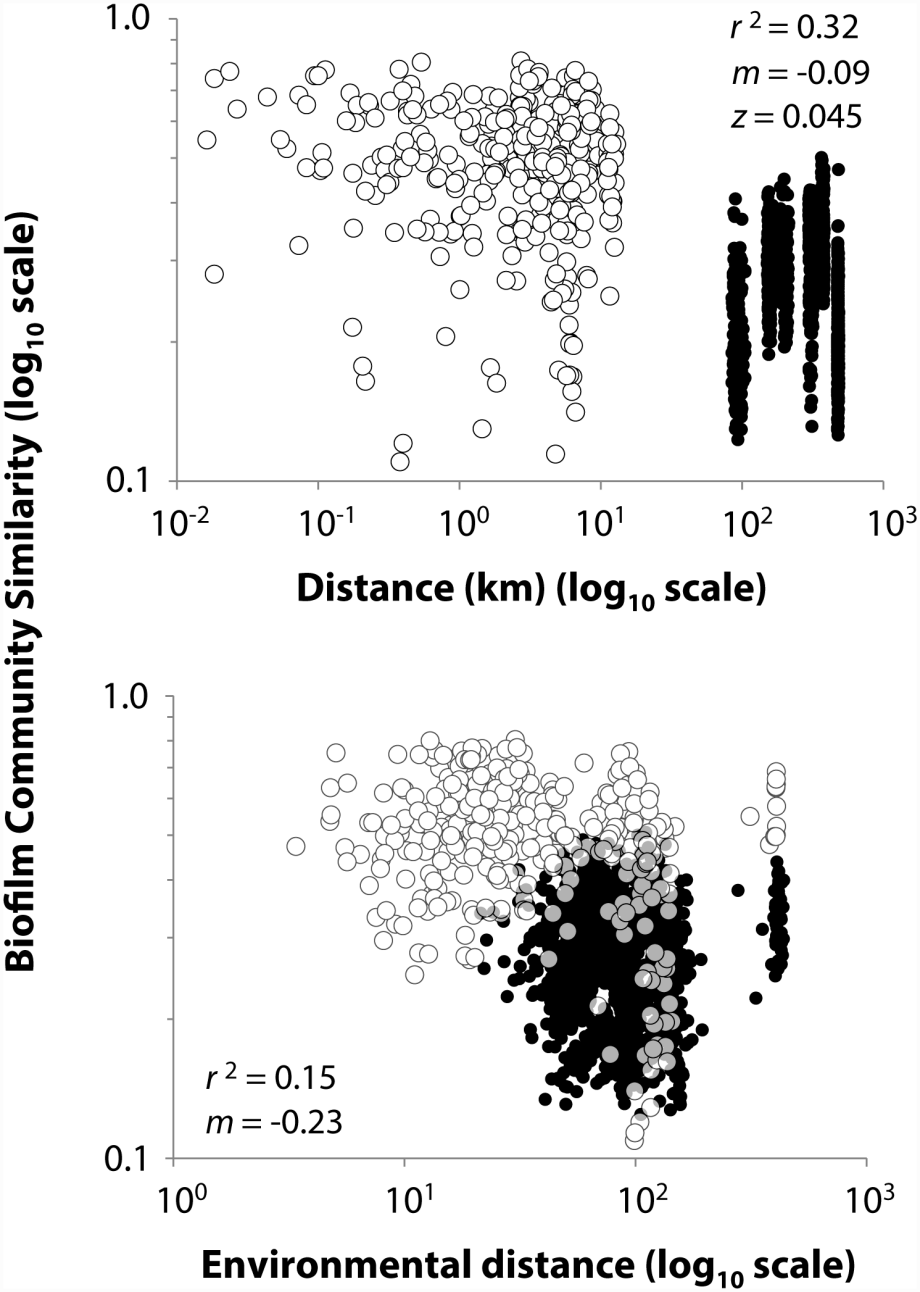
Distance-decay relationships of community similarity for epilithic biofilms. Data represent log-log regressions of Sørensen similarity index values calculated from OTUs abundances versus geographic (upper panel) and environmental (lower panel) distances for within floodplains (open dots) and among floodplains (filled dots). Both slopes (*m*) were significantly different from zero, *p*-value <0.01. Scaling coefficient (*z*) for the geographic distance-decay was derived from the plot as $z=\;-\frac{m}{2}$ following Harte et al. [41].

Significant declines in community similarity with spatial proximity, and also with environmental distance, suggests the existence of at least some geographic differentiation ([36]; Fig 6). The slope of the log-log relationship between community similarity and geographic distance (i.e., distance-decay relationship, *m* = -0.09) and the estimated scaling coefficient for OTU-area relationship (*z* = 0.045) inferred from it [40], were respectively similar to those observed for diatoms (*m* = -0.05 in Hillebrand et al. [41]) and ciliate protozoa (*z* = 0.043 in Finlay et al. [42]) of freshwater ecosystems. Slopes of distance-decay relationships tend to be lower (i.e., less differentiation per unit distance) for microorganisms than for larger organisms [18] reflecting the exceptionally dispersal characteristic of microbes, including hydrologic [15] and atmospheric vectors [43, 44]. The geographic influences we observed here, however, appear tied to regional-scale differences in environmental conditions. Geographic effects observed as a distance-decay relationship were not a significant influence on microbial communities assembly when environmental effects were fixed (Table 1; among all floodplains Partial Mantel test). Along with similarly strong values for Mantel correlations within and across floodplains (Table 1), results suggest that both regional (among floodplains) and local (among habitats) controls on diversity are environmentally-driven (Fig 6), with the largest degree sorting occurring at the floodplain level where its different entities displayed the greatest environmental variation. Some evidence exists, however, for strictly spatial control over species sorting as partial Mantel tests for cyanobacterial similarity revealed significant correlations with both geographic and environmental distances (Table 1).

Studies addressing the distinction between spatial and environmental influences on aquatic biodiversity have distinguished trends in similarity associated with body size and vagility [15] with relatively consistent emphasis on environmental distance for microbiota [45]. Results from our research suggest that river basins and associated floodplains may simply reflect very large scale ‘patches’ within which environmental conditions select for biofilm community composition. Historically, running water systems have been grouped in various ways including position in the drainage system [46, 47], hydrologic regime [48], character of material transport [49], and water chemistry [50]. Discerning how these features combine to present large-scale environmental variation relevant to aquatic microbial diversity will require embracing cross-system and downstream designs, but should lead to recognition of critical features organizing the composition and abundance of microbial biota robustly recognized to drive aquatic ecosystem function.

## Supporting Information

S1 Fig Redundancy analysis between environmental parameters and biofilm composition. (PDF)

S2 Fig A Venn diagram showing the OTUs detected by our survey in each river-floodplain system. (PDF)

S1 Table Description of proposed hierarchical levels in river-floodplain systems. (PDF)

S2 Table Variation in floodplain environmental conditions among the four river-floodplain systems. (PDF)

S3 Table Mapping file used for QIIME analyses. (PDF)

S4 Table Most dominant OTUs found in the four rivers-floodplain systems. (PDF)

S5 Table Mantel correlations between biofilm community structure and environmental variables. (PDF)
