# Supplementary material for: Spatial Patterns in Biofilm Diversity across Hierarchical Levels of River-Floodplain Landscapes
Source: PLoS One. 2015 Dec 2;10(12):e0144303. doi: 10.1371/journal.pone.0144303 (PMC4668062; doi:10.1371/journal.pone.0144303)

Figure S1 Redundancy analysis between environmental parameters and biofilm composition. The percent of total variance explained by the model (37%) associated with each axis is indicated. Color coding represents samples from the four different floodplains as in Figure 2. Grey dots correspond to OTUs. Scale of bottom and left axes is adjusted to biofilm samples, while upper and right axes scale is adjusted to OTUs distribution.

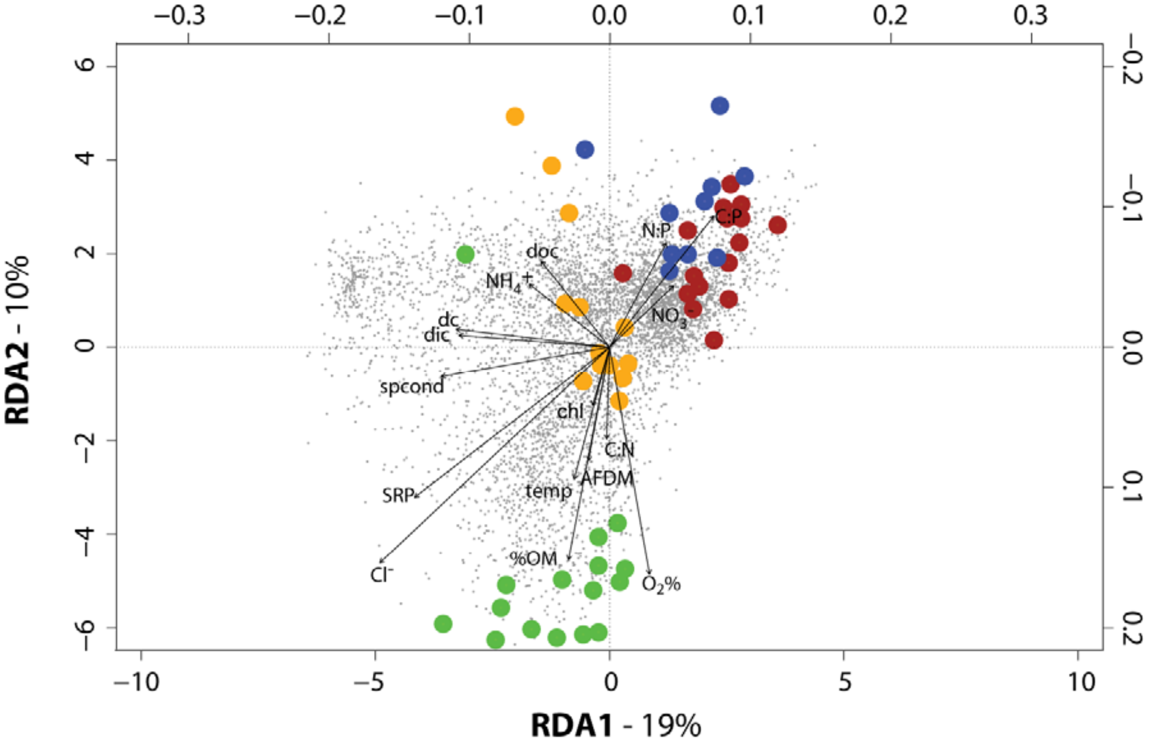

Supplement: S1 Fig — (PDF) [file pone.0144303.s001.pdf]
