# Supplementary material for: Spatial Patterns in Biofilm Diversity across Hierarchical Levels of River-Floodplain Landscapes
Source: PLoS One. 2015 Dec 2;10(12):e0144303. doi: 10.1371/journal.pone.0144303 (PMC4668062; doi:10.1371/journal.pone.0144303)

Figure S2 A Venn diagram showing the OTUs detected by our survey in each river-floodplain system. Percentages correspond to OTUs in each floodplain that were unique of that particular river system.

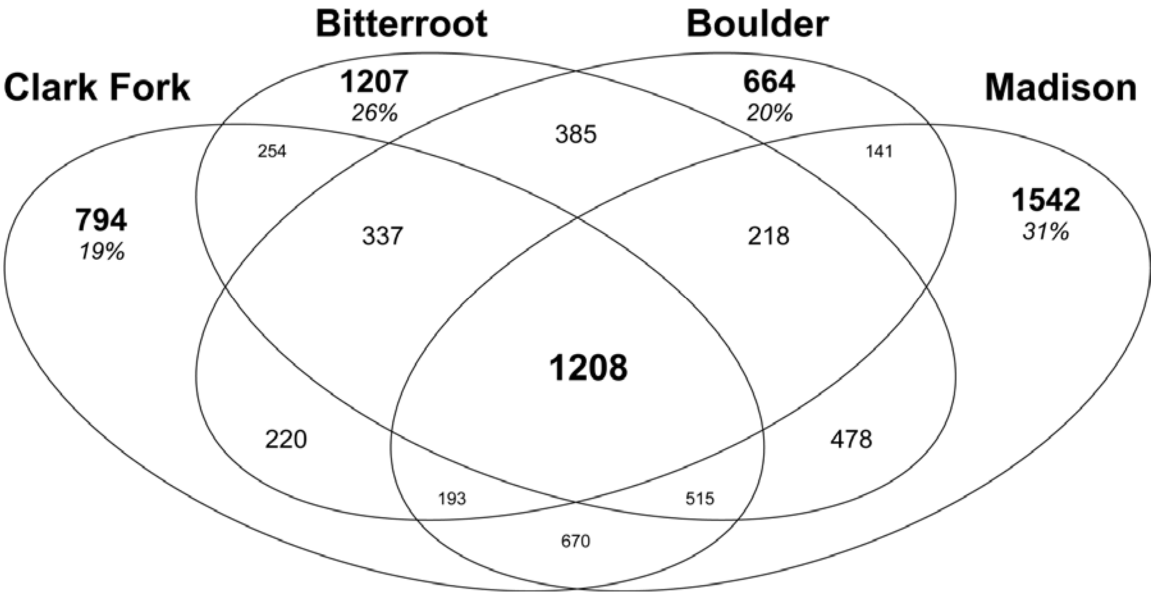

Supplement: S2 Fig — (PDF) [file pone.0144303.s002.pdf]
