# Supplementary material for: Spatial Patterns in Biofilm Diversity across Hierarchical Levels of River-Floodplain Landscapes
Source: PLoS One. 2015 Dec 2;10(12):e0144303. doi: 10.1371/journal.pone.0144303 (PMC4668062; doi:10.1371/journal.pone.0144303)
Supplement: S1 Table — (PDF) [file pone.0144303.s003.pdf]

Table S1 Description of proposed hierarchical levels in river-floodplain systems.

Level	Entity	Identity
Region	physiographic domain that defines geographic areas of generally similar climatic and hydrologic conditions	Columbia-Missouri headwaters, MT; all environmental space for all levels
Floodplain	geomorphic surfaces intimately associated with rivers, largest spatial entity at which discernable ecological features are identified	Clark Fork, Boulder, Bitterroot, and Madison River-floodplain systems; cumulative aquatic environments with a river-floodplain system
Zone	sub-systems reflecting spatial variation in aquatic-terrestrial interaction	main-channel and off-channel zones; environmental conditions reflecting changing autochthonous and allochthonous resource supply
Habitat	channel units that represent local environmental conditions and are the smallest scale at which environmental features are identified	main-channel habitats:<br>1) riffle – shallow, higher gradient, more turbulent flow<br>2) run – shallow, low-gradient, less turbulent flow<br>3) flow channel confluences – convergent flow nodes<br>4) shorelines – shallow near-shore areas<br><br>off-channel habitats:<br>1) side channels – backwaters and more isolated<br>2) ponds – lentic floodplain water<br>3) parafluvial springbrook – spring- fed stream within the scoured alluvial floodplain area<br>4) orthofluvial springbrook – spring-fed stream within more vegetated floodplain areas
Microhabitat	sub-habitat niche space that generates variation at the habitat scale	smallest scale of microbial habitat character that generates variation in niche space within channel units, unmeasured
