# Supplementary material for: Spatial Patterns in Biofilm Diversity across Hierarchical Levels of River-Floodplain Landscapes
Source: PLoS One. 2015 Dec 2;10(12):e0144303. doi: 10.1371/journal.pone.0144303 (PMC4668062; doi:10.1371/journal.pone.0144303)
Supplement: S2 Table — (PDF) [file pone.0144303.s004.pdf]

Table S2 Variation in floodplain environmental conditions among the four river-floodplain systems. Data are means ± standard error from all habitats sampled in each floodplain, and *p*-values are for Kruskal–Wallis tests (river as a factor). Means in a row with identical superscripts are not significantly different ($p < 0.05$).

Environmental parameters	Clark Fork	Boulder	Bitterroot	Madison	*p* -value
O_2 saturation (%)	84.6 ± 4.0	70.3 ± 3.4	78.2 ± 5.9	96.6 ± 5.9	0.158
Sp. Conductivity (μS/cm)	393.3 ± 7.4^{a}	162.8 ± 8.1^{b}	120.6 ± 7.4^{c}	251.0 ± 11.6^{d}	**<0.001**
Temperature (ºC)	18.0 ± 0.7	18.4 ± 0.6	19.8 ± 0.5	20.9 ± 0.8	0.052
TIC (ppm)	28.2 ± 1.0^{a}	13.9 ± 0.9^{b}	12.1 ± 0.4^{b}	17.5 ± 0.9^{c}	**<0.001**
DOC (ppm)	2.5 ± 0.1^{a}	3.1 ± 0.1^{b}	2.0 ± 0.1^{c}	2.3 ± 0.1^{a}	**<0.001**
TDC (ppm)	30.7 ± 1.0^{a}	17.0 ± 0.9^{b}	14.1 ± 0.4^{c}	19.8 ± 0.9^{b}	**<0.001**
Cl^- (ppm)	5.7 ± 0.3^{a}	3.8 ± 0.1^{b}	1.6 ± 0.1^{c}	15.6 ± 0.6^{d}	**<0.001**
NH_4^+ (ppb)	26.5 ± 18.9	6.9 ± 3.2	2.9 ± 0.9	12.2 ± 4.9	0.245
SRP (ppb)	39.2 ± 3.0^{a}	49.4 ± 1.1^{b}	12.1 ± 1.3^{c}	65.6 ± 1.6^{d}	**<0.001**
NO_3^- (ppb)	8.4 ± 6.1^{a}	5.8 ± 1.4^{ab}	22.1 ± 5.3^{b}	2.6 ± 0.6^{a}	**<0.001**
C/N	8.9 ± 1.8^{a}	2.6 ± 1.0^{ab}	1.9 ± 0.7^{b}	4.4 ± 0.9^{ab}	**<0.05**
N/P	1.5 ± 0.7^{a}	0.6 ± 0.1^{a}	4.4 ± 0.9^{b}	0.5 ± 0.2^{a}	**<0.05**
C/P	2.0 ± 0.1^{a}	0.7 ± 0.0^{bd}	2.9 ± 0.2^{c}	0.7 ± 0.0^{bd}	**<0.001**
AFDM (mg/cm^2)	8.6 ± 2.2^{a}	0.6 ± 0.2^{b}	1.0 ± 0.3^{b}	6.9 ± 1.5^{a}	**<0.05**
Organic matter (%)	27.6 ± 2.5^{ab}	36.9 ± 4.5^{ac}	19.6 ± 2.9^{bc}	51.9 ± 3.8^{c}	**<0.001**
Chlorophyll-*a* (mg/cm2)	158.2 ± 70.6^{a}	8.2 ± 2.2^{b}	13.4 ± 3.2^{b}	59.4 ± 10.4^{a}	**<0.001**

TDC=total dissolved carbon, SRP=soluble reactive phosphate. C/N, N/P, and C/P are molar ratios
