# Supplementary material for: Spatial Patterns in Biofilm Diversity across Hierarchical Levels of River-Floodplain Landscapes
Source: PLoS One. 2015 Dec 2;10(12):e0144303. doi: 10.1371/journal.pone.0144303 (PMC4668062; doi:10.1371/journal.pone.0144303)
Supplement: S3 Table — (PDF) [file pone.0144303.s005.pdf]

Table S3 Mapping file used for QIIME analyses. Data correspond to the barcode sequence used for each sample, the linker/primer sequence used to amplify the sample, and the environmental data (i.e., explanatory variables) for each biofilm sample.

Sample ID	Barcode Sequence	Linker Primer Sequence	river	zone	habitat	lat	lon	O_2 %	SpCond	T	TIC	DOC	TDC	Cl^-	NH_4^+	SRP	NO_3^-	C/N	N/P	C/P	AFDM	OM %	Chl-*a*
1	CATGTTGGAACA	CCGGACTACHVGGGTWTCTAAT	CF	MC	SR	46.6066	-113.0423	83.2	388.2	17.7	32.5	2.8	35.3	5.9	0.7	40.0	1.5	17.2	0.1	2.1	1.9	30.1	48.3
2	ATGGGACCTTCA	CCGGACTACHVGGGTWTCTAAT	CF	MC	RN	46.6088	-113.0455	104.9	385.1	19.9	28.0	2.9	30.9	5.3	0.7	47.7	1.5	14.8	0.1	1.5	6.3	34.3	134.6
3	GCTATTCCTCAT	CCGGACTACHVGGGTWTCTAAT	CF	MC	SL	46.6084	-113.0438	78.8	388.2	16.9	24.0	2.7	26.7	4.5	246.9	69.7	2.8	0.1	7.9	0.9	6.4	34.1	44.2
4	AGTGATGTGACT	CCGGACTACHVGGGTWTCTAAT	CF	OC	FC	46.6118	-113.0487	64.3	389.4	15.9	25.1	3.2	28.3	6.2	0.7	39.0	6.5	4.1	0.4	1.7	0.7	21.2	16.2
5	CTTAGCTACTCT	CCGGACTACHVGGGTWTCTAAT	CF	MC	SR	46.6242	-113.0844	78.8	393.5	16.9	27.3	2.5	29.8	6.5	0.7	29.7	1.5	14.4	0.2	2.4	22.1	26.6	38.3
6	CCTGACACACAC	CCGGACTACHVGGGTWTCTAAT	CF	MC	RN	46.6221	-113.0808	74.9	331.3	16.7	30.5	2.8	33.3	6.7	1.6	30.0	2.5	8.7	0.3	2.6	1.5	12.5	39.1
7	CACGTTTATTCC	CCGGACTACHVGGGTWTCTAAT	CF	MC	SL	46.624	-113.0845	76	393.1	16.8	21.6	2.6	24.2	6.2	0.7	36.0	1.5	11.5	0.1	1.5	5.2	23.0	59.2
8	CAGTTCGAGATA	CCGGACTACHVGGGTWTCTAAT	CF	OC	SB	46.6227	-113.09	58.1	458	13.1	35.5	1.5	37.0	3.1	29.2	42.0	81.8	0.4	5.8	2.2	8.9	30.9	488.3
9	AATGTCACCAGA	CCGGACTACHVGGGTWTCTAAT	BT	MC	SR	46.667	-114.039	94.7	125.2	20	12.3	1.7	14.0	2.3	0.5	11.0	2.7	4.5	0.6	2.9	0.0	49.6	0.1
10	CAGCCTGCAAAT	CCGGACTACHVGGGTWTCTAAT	BT	MC	RN	46.668	-114.0388	93.5	125.3	19.8	13.3	2.3	15.5	1.8	3.5	10.3	8.7	1.3	2.6	3.3	0.5	6.2	7.9
11	CAAGCGTTGTCC	CCGGACTACHVGGGTWTCTAAT	BT	MC	SL	46.6682	-114.039	89.4	117.5	19.9	12.1	1.8	13.9	1.4	0.5	10.7	18.3	0.7	3.9	2.9	0.5	17.5	11.4
12	GACTTATGCCCG	CCGGACTACHVGGGTWTCTAAT	BT	MC	CZ	46.6689	-114.0394	83.9	124.9	19.2	12.2	2.3	14.5	1.9	13.8	10.3	16.3	0.5	6.4	3.0	0.7	11.1	9.0
13	ATGTAGGCTTAG	CCGGACTACHVGGGTWTCTAAT	BT	OC	SB	46.6683	-114.0412	27.5	78.5	18	8.1	1.5	9.6	0.9	2.0	18.0	39.0	0.2	5.0	1.2	1.7	9.2	30.2
14	CGCGTCAAACTA	CCGGACTACHVGGGTWTCTAAT	BT	OC	SB	46.7147	-114.0411	37.2	101	18.3	14.2	1.3	15.5	1.3	1.6	28.3	51.0	0.3	4.1	1.3	0.1	37.9	0.7
15	GCCGAGATAATT	CCGGACTACHVGGGTWTCTAAT	BT	OC	FC	46.6644	-114.0535	106.2	114.3	23.5	11.8	1.8	13.5	1.5	3.7	10.7	1.0	2.9	1.0	2.8	3.6	21.6	38.8
16	AACATGCATGCC	CCGGACTACHVGGGTWTCTAAT	BT	MC	SR	46.6909	-114.0453	80	121	18.4	13.2	2.3	15.4	1.4	0.5	8.0	24.7	0.6	7.0	4.2	2.4	15.9	19.5
17	GCCCTATCTTCT	CCGGACTACHVGGGTWTCTAAT	BT	MC	RN	46.6955	-114.0474	89.4	122	19.4	13.0	2.3	15.2	1.7	1.1	9.3	21.7	0.7	5.4	3.6	0.4	20.3	5.5
18	AGGTACGCAATT	CCGGACTACHVGGGTWTCTAAT	BT	MC	SL	46.6906	-114.0459	82.3	2	18.8	11.9	1.9	13.8	1.6	0.5	11.7	14.7	0.9	2.9	2.6	0.3	18.3	6.0
19	GTCCCTATTATC	CCGGACTACHVGGGTWTCTAAT	CF	MC	SR	46.6361	-113.1036	92.8	394.1	18.1	31.1	2.4	33.5	6.2	0.7	33.7	1.5	16.5	0.1	2.4	20.7	35.1	156.0
20	AAGTATCCTGCG	CCGGACTACHVGGGTWTCTAAT	CF	MC	RN	46.6372	-113.1071	98.6	394	19.8	27.5	2.4	29.9	6.4	5.4	31.0	2.5	4.0	0.6	2.3	22.2	36.1	896.6
21	CAAATGGTCGTC	CCGGACTACHVGGGTWTCTAAT	CF	MC	SL	46.6345	-113.1059	93.7	389	19.2	29.2	2.4	31.6	6.1	0.7	40.0	1.5	15.5	0.1	1.9	5.8	25.2	82.6
22	AATCAACTAGGC	CCGGACTACHVGGGTWTCTAAT	CF	OC	SB	46.6369	-113.1089	98.1	399.1	22.4	27.3	2.0	29.3	5.6	55.3	30.7	1.5	0.6	4.1	2.3	3.4	40.2	25.0
23	ACGCATCGCACT	CCGGACTACHVGGGTWTCTAAT	CF	MC	CZ	46.6395	-113.1162	97.2	410.5	21	27.0	2.4	29.4	6.3	0.9	39.7	2.8	8.5	0.2	1.8	6.3	10.1	28.9
24	CACCGAAATCTG	CCGGACTACHVGGGTWTCTAAT	BO	MC	SL	46.1648	-112.0116	80.4	150.8	20.8	9.3	2.3	11.6	3.9	5.1	43.7	5.3	1.0	0.5	0.6	0.4	23.4	14.5
25	CTCGATGTAAGC	CCGGACTACHVGGGTWTCTAAT	BO	MC	RN	46.1568	-111.9978	74.8	163.9	17.6	9.9	3.8	13.7	3.3	9.3	48.7	2.3	1.0	0.5	0.5	4.4	29.9	87.6
26	AGCCAGTCATAC	CCGGACTACHVGGGTWTCTAAT	BO	MC	RN	46.1415	-111.9835	78.8	171.8	17	11.5	3.0	14.5	3.6	6.1	49.0	2.3	1.6	0.4	0.6	1.6	28.8	9.4
27	ATACGCATCAAG	CCGGACTACHVGGGTWTCTAAT	BO	MC	SL	46.1322	-111.9604	68.4	125.7	19.5	17.3	3.3	20.6	3.9	0.7	49.7	4.0	4.3	0.2	0.9	0.6	23.5	16.0
28	AGATGTCCGTCA	CCGGACTACHVGGGTWTCTAAT	BO	OC	FC	46.1322	-111.9556	64.4	188.4	17.8	13.9	3.0	16.9	3.5	0.7	47.0	15.3	1.0	0.8	0.8	0.1	38.8	1.3
29	AGGGTACAGGGT	CCGGACTACHVGGGTWTCTAAT	BO	MC	CZ	46.1674	-112.0153	76.6	161	21	12.4	3.2	15.6	3.4	26.6	49.3	5.7	0.4	1.4	0.6	0.1	11.8	1.2
30	AGAGTGCTAATC	CCGGACTACHVGGGTWTCTAAT	BO	MC	CZ	46.1435	-111.9858	84.5	174.5	19.8	13.2	2.9	16.1	4.4	4.0	48.3	9.3	1.2	0.6	0.7	0.5	11.3	22.1
31	GAGGTTCTTGAC	CCGGACTACHVGGGTWTCTAAT	BO	MC	RN	46.1321	-111.9601	71	194.9	18.4	16.5	3.2	19.7	4.4	0.7	50.7	1.0	11.3	0.1	0.8	0.3	20.1	7.7
32	AAGGGACAAGTG	CCGGACTACHVGGGTWTCTAAT	BO	OC	PN	46.1355	-111.9659	47.8	173.7	20.8	16.5	3.8	20.3	3.6	5.4	55.7	2.0	2.6	0.3	0.8	0.3	51.4	2.4
33	AGTGTCGATTCG	CCGGACTACHVGGGTWTCTAAT	BO	MC	CZ	46.1392	-111.9713	62.8	173.7	21.9	16.3	2.6	18.9	4.3	0.7	47.0	4.0	4.0	0.2	0.9	1.9	31.1	15.2
34	CACTAACAAACG	CCGGACTACHVGGGTWTCTAAT	BO	MC	SR	46.1314	-111.9446	60.7	115.4	17.4	16.4	3.0	19.4	4.5	6.4	48.3	3.3	2.0	0.4	0.9	0.1	60.0	0.9
35	CCTACCATTGTT	CCGGACTACHVGGGTWTCTAAT	BO	MC	SR	46.1413	-111.9835	76	171.9	16	12.2	3.1	15.2	3.3	1.8	46.7	8.7	1.4	0.5	0.7	0.5	44.8	3.3
36	GAGTCCGTTGCT	CCGGACTACHVGGGTWTCTAAT	BO	MC	SR	46.156	-111.9949	70.3	164.5	16	12.4	3.4	15.8	3.6	34.2	54.0	4.7	0.4	1.6	0.6	0.6	51.3	4.9
37	CACGACTTGACA	CCGGACTACHVGGGTWTCTAAT	BO	MC	SL	46.1436	-111.9857	84.9	171.1	20.2	12.6	2.9	15.5	4.0	7.6	49.0	11.0	0.8	0.8	0.7	1.1	26.6	21.2
38	CTTGGAGGCTTA	CCGGACTACHVGGGTWTCTAAT	BO	OC	SB	46.1563	-111.9952	28.1	123.1	14	23.5	3.3	26.8	2.0	32.2	94.7	21.3	0.5	1.3	0.6	1.0	24.0	5.0
39	ACGTGGTTCCAC	CCGGACTACHVGGGTWTCTAAT	BT	OC	PN	46.6824	-114.039	106.8	126.8	23	12.5	2.1	14.5	2.0	3.9	10.7	64.7	0.2	14.2	3.0	1.9	5.4	11.9
NegC_1	CGAGTTCATCGA	CCGGACTACHVGGGTWTCTAAT																					
40	AAGCAGATTGTC	CCGGACTACHVGGGTWTCTAAT	BT	MC	CZ	46.691	-114.0462	83.9	121.4	18.8	13.4	2.0	15.4	1.8	0.5	9.0	1.0	10.1	0.4	3.8	0.3	20.0	4.2
41	ACTATGGGCTAA	CCGGACTACHVGGGTWTCTAAT	BT	OC	SB	46.6825	-114.043	52.4	99.8	17.5	10.8	2.0	12.8	1.3	8.2	16.0	24.7	0.4	4.5	1.7	1.7	22.1	31.8
42	CAAACCTATGGC	CCGGACTACHVGGGTWTCTAAT	BT	MC	SR	46.7189	-114.0452	97	117	21.2	11.9	2.3	14.2	1.6	2.2	8.3	1.0	4.4	0.8	3.7	0.3	29.7	2.5
43	ATCGCTTAAGGC	CCGGACTACHVGGGTWTCTAAT	BT	MC	SL	46.7211	-114.0459	74.6	116.6	18	12.8	1.9	14.7	1.8	0.5	7.3	4.0	3.3	1.4	4.5	1.5	13.2	32.5
44	ACCATCCAACGA	CCGGACTACHVGGGTWTCTAAT	BT	OC	FC	46.7292	-114.0663	127.8	85.6	24.5	6.6	3.3	9.9	3.9	4.3	12.3	30.3	0.2	6.2	1.4	1.3	39.1	13.5
45	ACAGGAGGGTGT	CCGGACTACHVGGGTWTCTAAT	BT	OC	SB	46.7139	-114.0495	52.8	99	22.6	10.7	2.4	13.2	1.4	3.3	13.3	60.0	0.2	10.5	2.1	0.2	15.9	2.9

46	CCGAACGTCACT	CCGGACTACHVGGGTWTCTAAT	MA	OC	FC	45.7277	-111.5182	115.5	233.6	21	17.5	2.3	19.8	15.5	17.6	66.7	5.7	0.9	0.8	0.7	1.7	54.4	54.6
47	ACACCAACACCA	CCGGACTACHVGGGTWTCTAAT	MA	MC	CZ	45.7283	-111.5183	108.2	232.2	19.7	16.2	2.3	18.4	14.1	1.3	63.3	1.0	8.3	0.1	0.7	3.5	55.3	93.7
48	AACTGGAACCCT	CCGGACTACHVGGGTWTCTAAT	MA	MC	SR	45.7282	-111.5192	107.1	236.6	19.2	17.2	2.6	19.8	15.4	0.7	62.3	1.3	9.9	0.1	0.7	6.2	65.3	45.7
49	ATACTCGGCTGC	CCGGACTACHVGGGTWTCTAAT	MA	MC	RN	45.7257	-111.5212	101	237.5	18.6	17.2	2.4	19.6	16.2	9.4	63.7	4.7	1.4	0.5	0.7	0.8	64.2	14.0
50	ACGCTTAACGAC	CCGGACTACHVGGGTWTCTAAT	MA	MC	SL	45.7284	-111.5191	107.7	228.3	22.4	16.3	2.4	18.7	15.6	3.6	67.0	3.0	2.9	0.2	0.6	0.9	54.2	30.0
51	AGCTTACCGACC	CCGGACTACHVGGGTWTCTAAT	MA	MC	CZ	45.7752	-111.5178	101.5	237.9	19.8	11.5	1.9	13.4	14.6	11.1	74.7	1.3	1.1	0.4	0.4	19.2	61.7	51.6
52	AGGGCTATAGTT	CCGGACTACHVGGGTWTCTAAT	MA	OC	FC	45.7746	-111.5168	103.1	238.3	20.1	19.0	2.3	21.3	16.1	4.9	65.0	1.3	3.5	0.2	0.8	14.0	78.1	15.3
53	ATCTTGGAGTCG	CCGGACTACHVGGGTWTCTAAT	MA	OC	PN	45.7766	-111.5161	20.2	354.6	11.6	24.6	1.8	26.4	19.1	13.8	70.7	10.0	1.2	0.7	0.9	12.6	22.2	75.9
54	AGCACCGGTCTT	CCGGACTACHVGGGTWTCTAAT	MA	MC	SR	45.7905	-111.5116	108.3	236.4	21.3	18.2	2.4	20.6	16.1	0.7	64.7	1.0	12.2	0.1	0.7	15.4	70.0	92.3
55	GATACGTTCGCA	CCGGACTACHVGGGTWTCTAAT	MA	MC	RN	45.7921	-111.512	108.6	236	21.9	16.0	2.4	18.4	10.2	5.9	52.0	3.0	2.1	0.4	0.8	11.3	65.3	92.3
56	ATCTAGTGGCAA	CCGGACTACHVGGGTWTCTAAT	MA	OC	PN	45.7784	-111.5159	50.3	397	19.9	28.2	3.1	31.3	21.5	48.4	83.0	1.7	0.7	1.3	0.9	1.4	29.7	23.6
57	AGCGGCCTATTA	CCGGACTACHVGGGTWTCTAAT	MA	MC	SL	45.7855	-111.5137	109.8	234	22.5	16.9	2.4	19.3	16.1	2.5	64.3	1.0	5.7	0.1	0.7	1.7	28.2	57.3
58	GAGTTAGCATCA	CCGGACTACHVGGGTWTCTAAT	MA	MC	SR	45.8221	-111.5007	88.7	234.9	19.7	17.6	2.2	19.8	15.7	76.5	60.3	2.7	0.3	2.9	0.8	2.5	57.9	45.8
59	ACATACTGAGCA	CCGGACTACHVGGGTWTCTAAT	MA	MC	CZ	45.8192	-111.5016	105.5	234.63	25.7	14.0	2.3	16.3	16.0	2.6	65.0	1.0	4.6	0.1	0.6	6.4	52.3	189.5
60	AAGAGCAGAGCC	CCGGACTACHVGGGTWTCTAAT	MA	OC	FC	45.8125	-111.5026	108.9	234.8	25.4	15.3	2.4	17.7	15.2	1.4	64.7	1.0	7.5	0.1	0.6	3.1	40.8	14.4
61	ACCTTGACAAGA	CCGGACTACHVGGGTWTCTAAT	MA	MC	RN	45.816	-111.5052	99.3	224.6	23.1	16.5	2.3	18.8	14.1	6.9	65.7	3.3	1.9	0.3	0.6	15.3	46.8	57.9
62	ACACGACTATAG	CCGGACTACHVGGGTWTCTAAT	MA	MC	SL	45.8144	-111.5043	98.6	234.9	23.6	15.9	2.2	18.1	13.9	0.8	62.0	1.0	10.5	0.1	0.7	0.9	36.3	55.6
NegC_2	CTCACGCAATGC	CCGGACTACHVGGGTWTCTAAT																					

lat=Latitude, lon=Longitude, O_2%=Oxygen saturation, SpCond=Specific conductivity, T=Temperature, TDC=total dissolved carbon, SRP=soluble reactive phosphate, AFDM=ash-free dry mass, OM%=Percentage of organic matter, and Chl-*a*=Chlorophyll-*a* abundance. C/N, N/P, and C/P are molar ratios.
