# Supplementary material for: Spatial Patterns in Biofilm Diversity across Hierarchical Levels of River-Floodplain Landscapes
Source: PLoS One. 2015 Dec 2;10(12):e0144303. doi: 10.1371/journal.pone.0144303 (PMC4668062; doi:10.1371/journal.pone.0144303)
Supplement: S4 Table — (PDF) [file pone.0144303.s006.pdf]

Table S4 Most dominant OTUs found in the four rivers-floodplain systems. Relative (Rel) and cumulative (Cum) contribution to total sequences abundance observed in this study. Data for each river-floodplain system represent river-specific relative abundance for dominant OTUs. OTUs were classified up to class (c), order (o), family (f), genus (g), or species (s) level.

	Among all floodplains		Within floodplains				
**OTU ID**	**Rel (%)**	**Cum (%)**	**Clark Fork**	**Boulder**	**Bitterrot**	**Madison**	**taxon ID**
1	9.3	9.3	3.35	7.21	6.76	9.70	(g) *Rhodobacter*
5	3.3	12.6	3.49	0.08	10.79	0.10	(g) *Exiguobacterium*
19	2.9	15.5	3.12	2.63	7.38	0.75	(g) *Rhodobacter*
9719	2.6	18.1	2.74	2.17	1.72	3.07	(g) *Rhodobacter*
20	2.5	20.6	2.71	5.61	4.44	0.60	(g) *Zymomonas*
10	2.4	23.0	2.58	3.01	4.85	0.57	(o) *Sphingomonadales*
9	2.3	25.4	2.47	9.63	0.58	1.85	(g) *Phormidium*
8	2.0	27.4	2.18	0.01	0.00	0.02	(f) *Nostocaceae*
13	1.7	29.1	1.85	1.75	0.91	1.14	(o) *Rhizobiales*
18	1.5	30.6	1.56	0.01	0.00	0.69	(g) *Calothrix*
18601	1.3	31.9	1.38	1.87	2.28	0.34	(g) *Rhodobacter*
30	1.1	33.0	1.19	0.62	1.92	0.72	(g) *Luteolibacter*
54	1.0	34.0	1.06	0.88	2.28	0.24	(o) *Rhizobiales*
25	1.0	35.0	1.03	0.22	2.89	0.10	(g) *Anaerospora*
26668	0.9	35.9	0.96	0.00	0.00	0.46	(c) *Nostocophycideae*
27	0.8	36.7	0.89	0.43	2.37	0.13	(f) *Pirellulaceae*
34	0.8	37.6	0.89	5.00	0.00	0.04	(f) *Nostocaceae*
44	0.8	38.4	0.88	0.03	0.02	2.50	(g) *Sphingopyxis*
89	0.8	39.2	0.85	1.00	1.02	0.72	(s) *Methylotenera mobilis*
426	0.7	39.9	0.77	0.79	0.35	1.05	(g) *Rhodobacter*
22337	0.7	40.6	0.76	0.00	0.00	2.18	(f) *Rivulariaceae*
12203	0.7	41.3	0.71	0.00	0.03	2.09	(f) *Nostocaceae*
22899	0.6	41.9	0.69	1.11	0.67	0.25	(f) *Rhodobacteraceae*
59	0.6	42.6	0.68	0.73	0.52	0.44	(o) *Alteromonadales*
227	0.6	43.2	0.64	0.26	0.82	0.59	(f) *Comamonadaceae*
91	0.6	43.7	0.60	0.00	0.00	1.77	(f) *Cytophagaceae*
41	0.6	44.3	0.60	0.00	0.00	1.77	(f) *Saprospiraceae*
8831	0.6	44.8	0.60	1.10	0.43	0.23	(o) *Sphingomonadales*
56	0.5	45.4	0.57	0.00	0.00	1.71	(c) *Nostocophycideae*
802	0.5	45.9	0.51	0.01	0.01	1.52	(o) *Sphingomonadales*
