# Supplementary material for: Spatial Patterns in Biofilm Diversity across Hierarchical Levels of River-Floodplain Landscapes
Source: PLoS One. 2015 Dec 2;10(12):e0144303. doi: 10.1371/journal.pone.0144303 (PMC4668062; doi:10.1371/journal.pone.0144303)
Supplement: S5 Table — (PDF) [file pone.0144303.s007.pdf]

Table S5 Mantel correlations between biofilm community structure and environmental variables.

	Bacteria					Cyanobacteria				
	Clark Fork	Boulder	Bitterroot	Madison	All rivers	Clark Fork	Boulder	Bitterroot	Madison	All rivers
O_2 saturation	-0.182	**-0.461***	**-0.584*****	**-0.476****	**-0.308*****	-0.132	**-0.537***	**-0.349****	**-0.453***	**-0.206*****
Sp. Conductivity	**-0.721*****	0.151	**-0.329***	**-0.671*****	**-0.344*****	**-0.343***	0.149	-0.092	**-0.623****	**-0.474*****
Temperature	**-0.564****	**-0.386****	-0.184	-0.243	-0.013	**-0.336***	-0.062	**-0.308***	-0.218	-0.001
TIC	-0.034	-0.014	**-0.387***	**-0.436***	**-0.200*****	0.060	-0.019	-0.250	**-0.577****	**-0.330*****
DOC	**-0.397***	-0.178	**-0.286***	**-0.423***	**-0.171****	-0.216	-0.124	-0.242	**-0.445***	**-0.313*****
TDC	0.003	-0.037	-0.233	**-0.439***	**-0.201*****	0.092	-0.062	-0.070	**-0.574****	**-0.319*****
Cl^-	-0.365	-0.092	-0.154	**-0.403***	**-0.757*****	-0.179	0.159	0.030	**-0.469***	**-0.339*****
NH_4^+	-0.355	0.071	0.005	-0.221	-0.088	-0.153	0.089	0.046	-0.181	-0.021
SRP	-0.017	-0.220	**-0.551****	**-0.356***	**-0.504*****	-0.118	-0.109	-0.302	**-0.344***	**-0.236*****
NO_3^-	**-0.423***	0.259	-0.241	-0.223	-0.068	-0.211	0.236	**-0.386***	-0.254	-0.091
C/N	-0.055	0.104	0.179	0.152	0.023	-0.014	0.128	0.188	0.191	**-0.100****
N/P	-0.328	0.155	0.054	-0.245	-0.053	-0.158	0.224	-0.187	0.201	-0.030
C/P	-0.047	0.038	**-0.363****	**-0.326***	**-0.114***	-0.066	0.044	-0.130	**-0.396***	-0.061
AFDM	0.068	0.248	-0.162	-0.063	**-0.161****	-0.118	-0.363	-0.177	-0.070	**-0.193***
Organic matter %	-0.228	**-0.355***	-0.173	**-0.472****	**-0.292*****	-0.098	-0.008	0.021	**-0.414****	-0.038
Chlorophyll-*a*	-0.118	-0.029	-0.173	0.078	**-0.194****	-0.281	0.086	-0.081	-0.104	**-0.194****

*P-value<0.05, **P-value<0.01, ***P-value<0.001
